# Relationship between BDNF content in cord blood and early neurobehavior in newborns with subclinical hypothyroidism during pregnancy: a preliminary study

**DOI:** 10.3389/fneur.2024.1465715

**Published:** 2025-01-07

**Authors:** Hui Xu, Ya-Nan Du, Shuai Yang, Yu-Wen Guo

**Affiliations:** ^1^Department of Obstetrics, Affiliated Maternity and Child Health Hospital of Anhui Medical University, Hefei, China; ^2^Department of Neurocritical Care Unit, The First Affiliated Hospital of USTC, Division of Life Sciences and Medicine, University of Science and Technology of China, Hefei, China

**Keywords:** subclinical hypothyroidism during pregnancy, neurobehavioral capabilities, brain-derived neurotrophic factor, neonatal behavioral neurological assessment, newborns

## Abstract

**Objectives:**

Research on neurobehavioral abnormalities in neonates of mothers with subclinical hypothyroidism (SCH) is limited. The link between umbilical cord blood brain-derived neurotrophic factor (BDNF) levels and neurobehavioral outcomes in neonates has not been explored. This study investigates the correlation between alterations in umbilical cord blood BDNF levels and early neurobehavioral abnormalities in neonates born to pregnant women with SCH.

**Methods:**

This study recruited 72 pregnant women with SCH and 76 healthy controls (HC). The study collected general information for all subjects, including body mass index, parity, thyroid function assessed during early to late pregnancy, and neonatal birth weight. Neonatal behavioral and neural abilities were evaluated using the Neonatal Behavioral Neurological Assessment (NBNA). BDNF levels in umbilical cord blood were measured using the Enzyme-Linked Immunosorbent Assay method.

**Results:**

The results indicated that neonates with SCH during pregnancy had lower total NBNA scores, behavioral ability, passive muscle tone, active muscle tone, primitive reflexes, general assessment, and lower levels of cord blood BDNF compared to healthy controls. The cord blood BDNF of newborns with SCH during pregnancy was positively correlated with total NBNA score, behavioral ability, active muscle tone, and general assessment. Moreover, multiple linear regression analysis demonstrated an association between cord blood BDNF levels in pregnant patients with SCH and multiple measures of newborn health, including total NBNA score, behavioral ability, active muscle tone, and general assessment.

**Conclusion:**

Infants born to pregnant women with SCH exhibit reduced behavioral and neural abilities linked to BDNF levels in umbilical cord blood.

## Introduction

Subclinical hypothyroidism (SCH) during pregnancy is a type of pregnancy-related thyroid dysfunction characterized by a high serum level of thyroid-stimulating hormone (TSH) above the pregnancy-specific reference range, while the level of serum-free thyroxine (FT4) remains within the pregnancy-specific reference range (1). Studies have indicated that subclinical hypothyroidism during pregnancy is associated with a higher risk of adverse pregnancy outcomes, such as spontaneous abortion, preterm delivery, neonatal asphyxia, and neonatal death (2, 3).

Although SCH during pregnancy has received increasing attention from clinicians regarding its impact on pregnancy outcomes, there is still no consistent conclusion on whether SCH during pregnancy affects the offspring’s neurodevelopment. Williams demonstrated in a study of cognitive behavior in children aged around 5 years that for every 1 mL increase in maternal serum TSH levels during pregnancy, the score on the general cognitive index decreased by 3.2 points (4). Liu found that the offspring of mothers with SCH performed worse on the Morris water maze test than normal controls (5). However, some studies have discovered no association between maternal SCH and the mental deficits seen in the offspring (6). The conflicting results of previous studies may be attributed to variations in the TSH cutoff values used between studies. In addition, previous research on cognitive function in the offspring of SCH during pregnancy has focused on childhood, even as they approach adulthood. Giulia Bramati demonstrated that environmental enrichment increases complex spatial learning abilities and leads to long-lasting morphological changes in the hippocampus (7). Exercise has also been found to promote synaptic plasticity in the hippocampus and improve cognitive functions such as learning memory (8). Therefore, it is essential to minimize the adverse interference of the acquired environment on the neonate and to emphasize the influence of the intrauterine environment on the cognitive level of the offspring. This study evaluated the behavioral and neurological abilities of newborns with gestational SCH within 3 days of birth to minimize the influence of the acquired environment on the experimental results.

The Neonatal Behavioral Neurological Assessment (NBNA) is a widely used behavioral neurological assessment tool in neonatal medicine designed to assess neonatal behavioral neurology’s developmental level and functional status (9). Although the validity of the NBNA scale has been extensively demonstrated, scale testing requires extensive training, its results are highly subjective, and assessment is time-consuming and labor-intensive. Therefore, there is a great need for clinicians to have a more accessible and objective indicator of the behavioral and neurological abilities of newborns. Hematologic indicators are easily accessible, and monitoring instruments are well-established, convenient, and practical, making them highly promising as objective indicators for assessing the neurobehavioral abilities of neonates.

Brain-derived neurotrophic factor (BDNF) is currently the most comprehensively researched neurotrophic factor, which is primarily expressed in the central nervous system and is involved in many brain functions, including neuronal survival, axonal growth, cell migration, regulation of excitatory-inhibitory balance, and glutamate-dependent dendritic growth, synapse formation, stabilization, and plasticity (10, 11). BDNF plays a crucial role in hippocampal neurogenesis, experience-dependent neural plasticity, neuronal formation, and survival (12, 13). While markers such as S100B and NSE are commonly used to assess glial injury and neuronal damage, their primary roles are associated with pathological conditions, such as hypoxic–ischemic encephalopathy or traumatic brain injury (14). In contrast, BDNF is a key neurotrophic factor involved in normal neurodevelopment and has been specifically linked to cognitive and behavioral outcomes. Given the exploratory nature of this study, BDNF was prioritized as a biomarker for its direct relevance to the neurodevelopmental processes under investigation. Numerous animal experiments and clinical trials have demonstrated that BDNF may be essential to cognitive processes. For example, research has indicated that thyroid hormones may induce morphological changes in the hippocampus by upregulating BDNF (15). Furthermore, the previous research conducted by our research group has revealed that the levels of BDNF in patients with insomnia are significantly reduced, which is notably associated with cognitive function (16).

During fetal development, changes in thyroid function may affect the expression of BDNF in the hippocampus of offspring, leading to structural and functional dysplasia of the central nervous system. Abdelhaffez observed a decrease in BDNF levels in the offspring of rats with Methimazole induced pregnancy-induced hypothyroidism. BDNF showed a negative correlation with TSH levels and a positive correlation with FT4 levels (17). Shafiee found that the reduction of BDNF in the hippocampus is associated with impaired spatial learning and working memory of offspring born to mothers with pregnancy-induced hypothyroidism (18). Although many studies have found a close relationship between thyroid function and BDNF, previous research has mainly focused on patients with pregnancy-induced hypothyroidism. There is limited research on the impact of SCH on BDNF during pregnancy, and the experimental findings are inconclusive. Therefore, it is necessary to conduct more research to explore the relationship between pregnancy-induced hypothyroidism and BDNF, and the neurodevelopment of offspring, especially in clinical research.

In this study, we hypothesize that newborns of pregnant women with SCH exhibit a decrease in BDNF levels in umbilical cord blood compared to normal controls and that this decrease is linked to the early behavioral and neurological abilities of the newborn. This study is the first to explore the relationship between early neurobehavioral abilities and cord blood BDNF levels in pregnant women with SCH, to provide clinical evidence for diagnosing and treating cognitive impairments related to SCH.

## Materials and methods

### Subjects

The study recruited expectant mothers from the Affiliated Maternity and child health Hospital of Anhui Medical University. All participants in the study satisfied the diagnostic criteria for gestational SCH outlined by the Guidelines for the Diagnosis and Treatment of Thyroid Diseases in Pregnancy and Postpartum (2nd edition), published by the Chinese Medical Association Obstetrics and Gynecology Branch (1). Subjects with hyperthyroidism, thyroid tumors, or other thyroid disorders were excluded from the study. Healthy pregnant women from the same hospital were recruited during the same period as the control group. The study comprised 81 cases of gestational SCH, of which 5 gestational SCH patients refused to sign the informed consent form and 4 patients declined to undergo neurobehavioral assessments. The study group comprised 72 patients, while the control group had 76 participants. The Obstetrics and Gynecology Hospital Ethics Committee affiliated with Anhui Medical University approved this experiment (No. YYLL2019-2019xlj17-02-01). All participants provided informed consent.

### Baseline data collection

Basic information on pregnant women, such as age, height, weight, health condition, parity, adverse obstetric history, previous pregnancy details, and medical history, were obtained through face-to-face interviews.

### Thyroid function and cord blood BDNF

Chemiluminescent microparticle immunoassay was used to determine thyroid peroxidase antibodies (TPOAB), TSH, and FT4 levels in the blood. The level of BDNF in cord blood was assessed using Enzyme-Linked Immunosorbent Assay.

### NBNA scale

The NBNA scale was used to assess the neurobehavioral capabilities of newborns (9). This assessment tool consists of five clusters: behavioral capacity (6 items), passive muscle tone (4 items), active muscle tone (4 items), primitive reflexes (3 items), and general assessment (3 items). Each item is assessed using a scale of 0 to 2, with a total possible score of 40. A score greater than 37 indicates an excellent developmental profile, whereas a score below 35 indicates abnormal neurobehavioral development. Scoring was conducted by experienced pediatricians who were blinded to maternal clinical data and group assignments.

### Statistical analysis

The statistical analysis was performed using SPSS 22.0. The results were presented as X ± S for normally distributed continuous variables, and independent sample t-tests were performed for intergroup comparison. Non-normally distributed variables were analyzed using median (interquartile range) [M (Q1, Q3)], and the Mann–Whitney-*U* test was used for intergroup comparison. Spearman’s rank correlation was used to analyze the correlation between BDNF and maternal thyroid function levels during pregnancy and neonatal behavioral neurology. Additionally, multiple linear regression analysis was conducted to investigate the relationship between BDNF, TSH, and cognitive function. A *p*-value less than 0.05 was considered statistically significant.

## Results

### General information and clinical data

No significant differences in age, parturition times, total stage of labor, birth weight, fetal sex, and mode of delivery were observed between the pregnant women with subclinical hypothyroidism (SCH) and the healthy control group (*p* > 0.05). The gestational age of the SCH group was lower than that of the healthy control group (*t* = 2.003, *p* < 0.05). The pre-pregnancy BMI (*Z* = −2.224, *p* < 0.05) and pre-delivery BMI (*t* = −1.992, *p* < 0.05) were higher in the SCH group than in the healthy control group. The first stage of labor (*Z* = −2.039, *p* < 0.05) and the second stage of labor (*Z* = −2.343, *p* < 0.05) were longer in the SCH group than in the healthy control group (Table 1).

**Table 1:** Comparison of general data and clinical information between the two groups.

	HC	SCH	*t*/*z*	*p*	95% CI
Age	29.9 ± 3.9	29.4 ± 3.9	0.745	0.457	−0.79, 1.75
Pre-pregnancy BMI	20.6 [19.2, 22.9]	21.5 [20.4, 24.1]	−2.224	0.025	−1.85, −0.11
Pre-delivery BMI	26.7 ± 3.2	27.4 ± 2.9	−1.992	0.048	−2.03, −0.01
Gestation times	2 [1, 3]	2 [1, 3]	−0.239	0.811	0.00, 0.00
Parturition times	1 [1, 2]	1 [1, 2]	−0.624	0.532	0.00, 0.00
Maternal type (primary/menstrual)	46/30	48/24	0.602	0.438	
History of pregnancy and birth (none/presence)	76/0	71/1	1.0623	0.487	
Gestational age	39.6 ± 1.1	39.2 ± 0.8	2.003	0.047	0.004, 0.625
Mode of delivery	67/9	62/10	0.138	0.710	
Fetal sex (male/female)	50/26	37/35	3.165	0.075	
Birth weight	3350.4 ± 405.7	3238.7 ± 355.1	1.778	0.077	−12.4, 235.9
Total stage of labor	4.8 [3.2, 10.3]	6.0 [4.3, 9.4]	−1.796	0.073	−2.23, −0.10
The first stage of labor	4.0 [2.5, 10.0]	5.6 [4.0, 9.0]	−2.039	0.041	−2.30, −0.13
The second stage of labor	0.5 [0.3, 0.7]	0.6 [0.4, 0.8]	−2.343	0.019	−0.23, −0.06

BMI, body mass index; HC, healthy controls; SCH, subclinical hypothyroidism during pregnancy.

### Comparison of thyroid function during early, middle, and late pregnancy in two groups of participants

The pregnant women with SCH exhibited significant differences in thyroid function in early, middle, and late pregnancy compared to the healthy control group. The TSH level during the early and middle pregnancy of the SCH group was higher than that of the healthy control group (*p* < 0.001). During late pregnancy, the TPOAb level (*Z* = −2.878, *p* < 0.01) and TSH level (*Z* = −5.925, *p* < 0.001) of the SCH group were higher than those of the healthy control group. No significant differences were observed in the other thyroid function levels (Table 2).

**Table 2:** Comparison of thyroid function in the early, middle, and third trimesters of pregnancy between the two groups.

Variable	HC	SCH	*t*/*z*	*p*	95% CI
TPOAB_E_	8.7 [6.6, 9.5]	6.8 [5.8, 8.5]	−1.800	0.072	−1.540, 0.090
TSH_E_	1.5 [1.3, 1.9]	4.3 [4.1, 4.8]	−10.504	<0.001	−3.03, −2.72
FT4_E_	1.0 [0.9, 1.0]	1.0 [0.9, 1.0]	−0.766	0.444	−0.03, 0.01
TPOAB_M_	8.7 [6.6, 9.7]	8.0 [6.1, 9.6]	−1.04	0.298	−0.44, 1.19
TSH_M_	1.5 [1.3, 1.8]	3.3 [2.0, 3.7]	−8.453	<0.001	−1.95, −1.30
FT4_M_	1.0 [0.9, 1.0]	1.0 [0.9, 1.0]	−1.356	0.175	−0.01, 0.06
TPOAB_L_	8.0 [6.8, 9.3]	9.6 [7.2, 13.7]	−2.878	0.004	−2.94, −0.51
TSH_L_	1.6 [1.5, 1.9]	2.4 [1.7, 3.8]	−5.925	<0.001	−1.26, −0.58
FT4_L_	0.9 [0.8, 1.0]	1.0 [0.9, 1.0]	−1.929	0.054	−0.09, 0.00

BMI, body mass index; _E_, early trimesters of pregnancy; FT4, free thyroxine; HC, healthy controls; _L_, third trimesters of pregnancy; _M_, middle trimesters of pregnancy; SCH, subclinical hypothyroidism during pregnancy; TPOAB, thyroid peroxidase antibody; TSH, thyroid stimulating hormone.

### Comparison of neonatal behavioral neurologic assessment scores and umbilical cord blood BDNF levels between two groups of subjects

**Figure 1:**
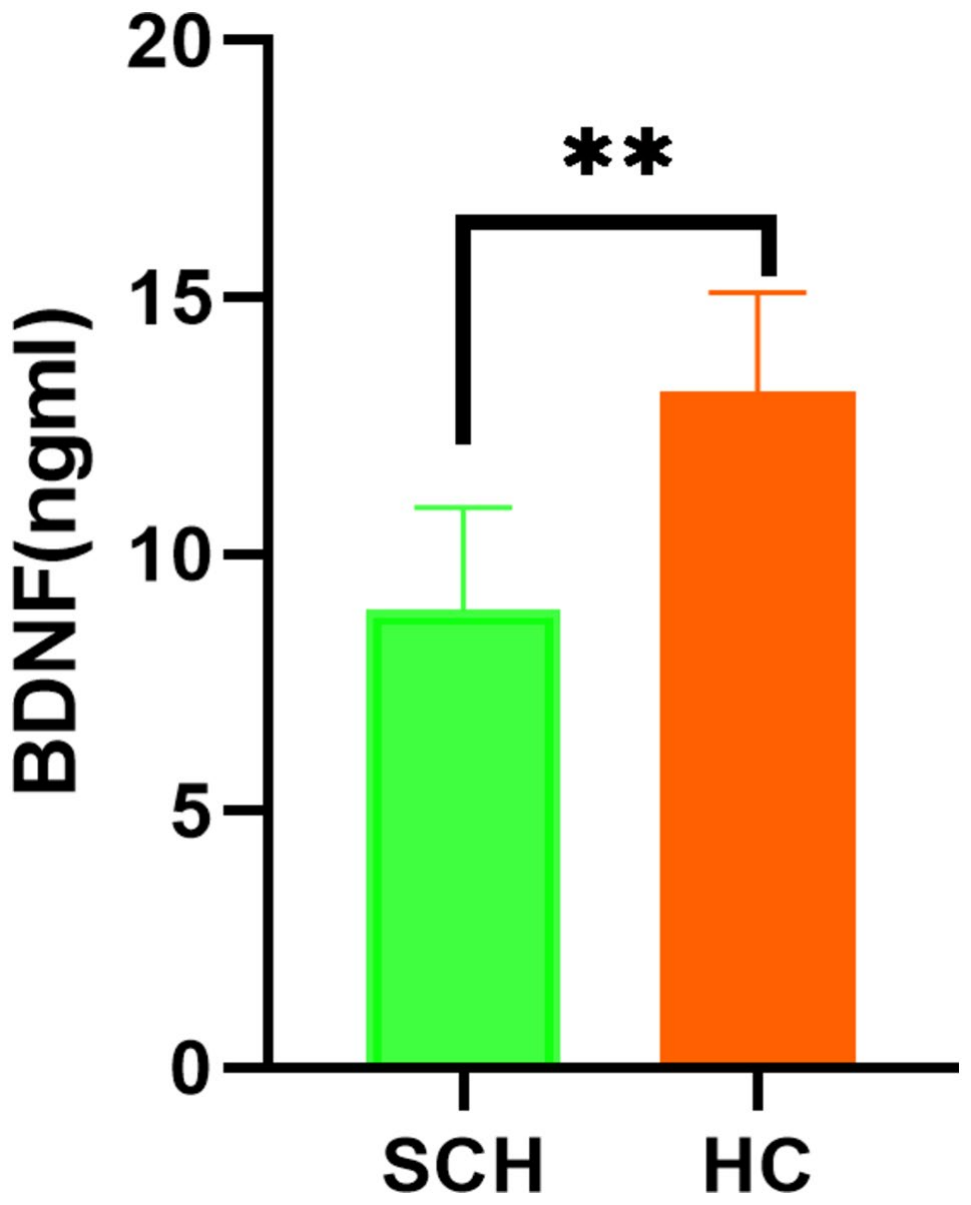
Comparison of the level of BDNF in the two groups. BNDF, brain-derived neurotrophic factor; HC, healthy controls; SCH, subclinical hypothyroidism during pregnancy. ** *p* < 0.01.

Newborns of the SCH group during pregnancy demonstrate lower total scores (*t* = 6.14, *p* < 0.001) on NBNA evaluation, as well as lower scores in behavior capacity (*t* = 2.48, *p* < 0.05), passive muscle tension (*t* = 3.19, *p* < 0.01), active muscle tension (*t* = 4.56, *p* < 0.001), primitive reflexes (*t* = 2.61, *p* < 0.05), and general assessment (*t* = 2.61, *p* < 0.05) as compared to the healthy control group. Furthermore, newborns in the SCH group had significantly lower levels of BDNF in their umbilical cord blood (*t* = 13.212, *p* < 0.001) (Table 3 and Figure 1).

**Table 3:** Comparison of neonatal neurobehavioral abilities in the two groups.

variable	HC	SCH	*t*	*p*	95% CI
NBNA	39.9 ± 0.2	39.3 ± 0.9	6.14	<0.001	0.46, 0.89
BC	12.0 ± 0.0	11.9 ± 0.3	2.48	0.014	0.02, 0.18
PMT	7.9 ± 0.2	7.8 ± 0.4	3.19	0.002	0.06, 0.25
AMT	7.9 ± 0.2	7.7 ± 0.5	4.56	<0.001	0.14, 0.36
PR	6.0 ± 0.0	5.92 ± 0.27	2.61	0.010	0.02, 0.14
GA	6.0 ± 0.0	5.92 ± 0.28	2.54	0.013	0.02, 0.15

AMT, active muscle tone; BC, behavioral capacity; BNDF, brain-derived neurotrophic factor; GA, general assessment; NBNA, the Neonatal Behavioral Neurological Assessment; PMT, passive muscle tone; PR, primitive reflexes; LHC, healthy controls; SCH, subclinical hypothyroidism during pregnancy.

### The correlation between the umbilical cord blood BDNF levels of newborns of pregnant women with SCH during different pregnancy stages (early, middle, and late) and thyroid function and cognitive function

**Figure 2:**
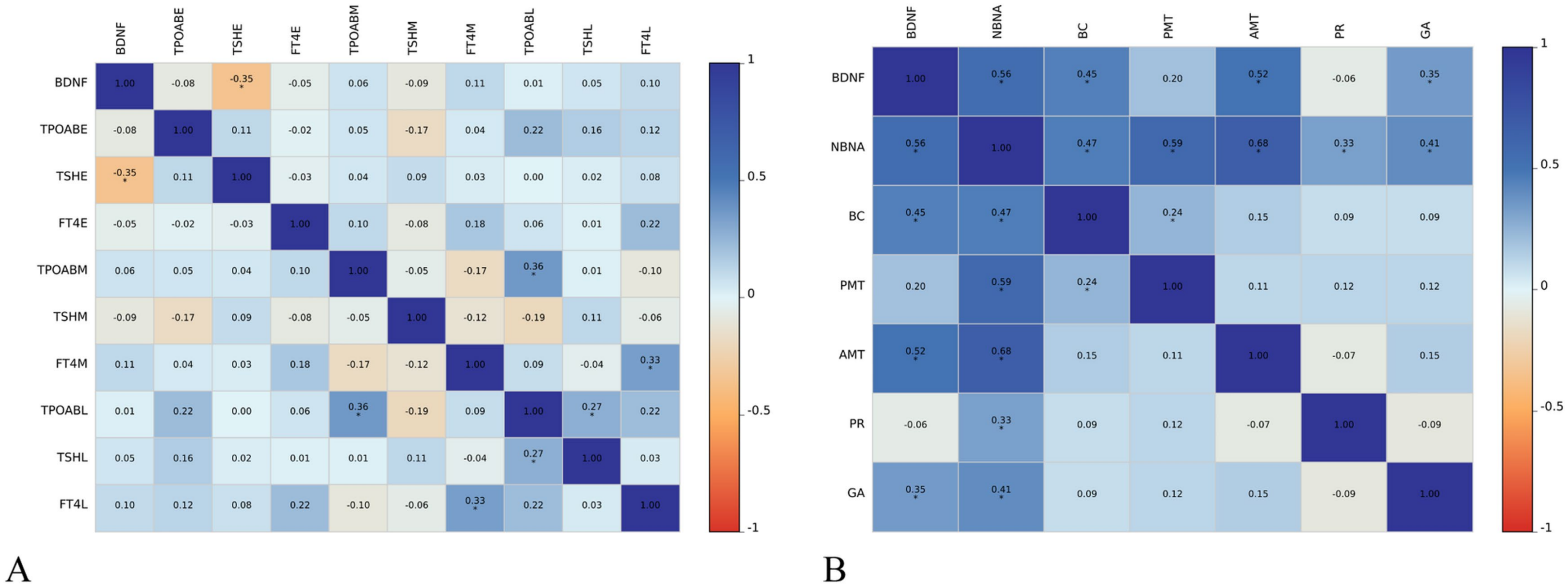
Relationship between cord blood BDNF level and thyroid function **(A)** and neonatal neurobehavioral abilities **(B)** in pregnancy in subclinical hypothyroidism group AMT, active muscle tone; BC, behavioral capacity; _E_, early trimesters of pregnancy; FT4, free thyroxine; GA, general assessment; _L_, third trimesters of pregnancy; _M_, middle trimesters of pregnancy; NBNA, the Neonatal Behavioral Neurological Assessment; PMT, passive muscle tone; PR, primitive reflexes; TPOAB, thyroid peroxidase antibody; TSH, thyroid stimulating hormone; HC, healthy controls; SCH, subclinical hypothyroidism during pregnancy. The color scale depicts the *r* range from −1 to 1.

The level of BDNF in the umbilical cord blood of newborns with SCH during pregnancy is negatively correlated with TSH levels in early pregnancy (*r* = −0.35, *p* < 0.01). However, no significant correlation is observed between BDNF levels and TPOAb and FT4 levels in early pregnancy, as well as thyroid function in middle and late pregnancy. In SCH patients during pregnancy, the level of BDNF in newborn umbilical cord blood is positively correlated with the NBNA total score within 3 days of birth (*r* = 0.56, *p* < 0.001), as well as behavior capacity (*r* = 0.45, *p* < 0.001), active muscle tension (*r* = 0.52, *p* < 0.001), and general assessment (*r* = 0.35, *p* < 0.01) of newborns. However, there is no significant correlation between BDNF level and passive muscle tension or primitive reflexes of newborns (Figure 2).

Subsequent multiple linear regression analysis (control age and birth weight) revealed that the BDNF level in pregnant women with SCH was positively associated with NBNA total score (*β* = 0.49, *t* = 4.26, *p* < 0.001), behavioral capacity (*β* = 0.32, *t* = 2.71, *p* < 0.01), active muscle tension (*β* = 0.54, *t* = 4.36, *p* < 0.001), and general assessment (*β* = 0.34, *t* = 2.54, *p* < 0.01). Conversely, TSH level showed a negative association with behavioral capacity (*β* = −0.35, *t* = −3.10, *p* < 0.01) in pregnant women with SCH (Table 4).

**Table 4:** Impact of BDNF and thyroid function in the multiple linear regression analyses in subclinical hypothyroidism group.

	BDNF			TSHE			Age			BW		
Independent variable	*β*	*t*	*p*	*β*	*t*	*p*	*β*	*t*	*p*	*β*	*t*	*p*
NBNA	0.49	4.26	0	−0.22	−2.00	0.50	0.23	0.23	0.82	0.02	0.20	0.85
BC	0.32	2.71	0.009	−0.35	−3.10	0.003	−0.09	−0.87	0.39	−0.16	−1.57	0.12
PMT	0.04	0.32	0.75	−0.18	−1.34	0.18	−0.00	−0.02	0.99	0.27	2.26	0.03
AMT	0.54	4.36	0	−0.01	−0.08	0.93	0.10	0.91	0.37	−0.04	−0.35	0.73
PR	0.06	−0.40	0.69	−0.09	−0.67	0.51	−0.11	−0.89	0.38	−0.17	−1.36	0.18
GA	0.34	2.54	0.01	0.06	0.43	0.67	0.14	1.20	0.24	0.12	0.99	0.33

AMT, active muscle tone; BA, behavioral capacity; BNDF, brain-derived neurotrophic factor; BW, Birth weight; _E_, early trimesters of pregnancy; GA, general assessment; NBNA, 20-item neurological examination; PMT, passive muscle tone; PR, primitive reflexes; TSH, thyroid stimulating hormone; HC, healthy controls; SCH, subclinical hypothyroidism during pregnancy.

## Discussion

Brain development from the fetal period to early life is crucial for various neurological and psychological domains throughout an individual’s life (19). This is the first study to investigate the relationship between the behavior and neural capacity of newborns in pregnant women with SCH and the level of BDNF in umbilical cord blood. Our study reveals that newborns’ early behavior and neural capacity in pregnant women with SCH were reduced and strongly correlated with the level of BDNF in umbilical cord blood.

The impact of SCH on pregnancy outcomes is relatively well established. However, the effect of SCH on the cognitive development of offspring remains inconclusive. A previous cohort study in the Netherlands involving 3,659 children and their mothers failed to establish a correlation between maternal TSH levels during early pregnancy and the cognitive development of offspring at 18 and 30 months (20). Similarly, a population-based cohort study in Spain, with 1,761 children and their mothers, failed to demonstrate any link between maternal serum TSH levels during pregnancy and neurodevelopment in children (21). We assessed newborns’ behavioral and neurological abilities in the SCH and normal control groups using the NBNA scoring method. Our results demonstrated that newborns in the SCH group had significantly lower total NBNA scores compared to those in the normal control group, and this difference was also observed in each section score. The inconsistent conclusions regarding the relationship between SCH during pregnancy and offspring neurodevelopment may be caused by varying cutoff values of TSH used in different studies, which could result in differences in the subjects included and lead to inconsistent results. Second, the age ranges of the study subjects differed substantially. Both the Dutch and Spanish studies focused on cognitive outcomes at 18 months or later, while our study assessed newborns within 3 days of birth. This early evaluation minimizes the influence of postnatal factors, such as education, social environment, or diet, which are known to affect cognitive development (22–24). Studies involving children and adult offspring often face confounding variables, possibly leading to biased outcomes. Our study evaluated the early behavioral and neurological abilities of newborns within 3 days after birth using the NBNA scale, which minimized interference from other factors, such as stress, and facilitated a more precise reflection of intrauterine effects on neonates. Future research should focus on standardizing SCH diagnostic criteria and incorporating longitudinal designs to better capture the long-term effects of maternal thyroid function on offspring neurodevelopment.

BDNF is a crucial protein molecule for neuronal growth, development, plasticity, and maintenance of the nervous system function (25). The binding of BDNF to the TrkB receptor triggers the activation of multiple signaling pathways, which regulate cellular biological processes, including cell proliferation, synaptic formation, and cell survival (26). Studies have shown that BDNF is essential in neural development, memory formation, learning, and neurological diseases (27). Abnormal regulation of BDNF levels is associated with various neurological disorders, such as Parkinson’s disease, anxiety, depression, and Alzheimer’s disease (28–30).

We observed a positive correlation between the levels of BDNF in newborn serum and their neurodevelopmental behavioral abilities, particularly with NBNA total score, passive muscle tone, and general assessment in the SCH group during pregnancy. Subsequent multivariate linear regression also revealed that BDNF levels in pregnant women with SCH were significantly associated with NBNA total score, active muscle tone, and general evaluation of behavioral abilities. Our research results suggest that BDNF may be involved in the pathological process of abnormal neurodevelopment in the offspring of mothers with SCH during pregnancy. Insufficient induction of high expression of BDNF in pregnant women with SCH may limit the behavioral neurodevelopment of fetuses during gestation, resulting in lower NBNA scores in newborns. BDNF, as a biological indicator, is more objective for evaluating newborn behavioral neurodevelopmental abilities than scales. However, these findings are preliminary, and the sensitivity, specificity, and predictive value of BDNF as a clinical diagnostic marker need further validation. Given the limitations of correlation analysis in establishing causality, future longitudinal and prospective studies should focus on pregnant women with SCH to better elucidate the relationships between maternal thyroid function, BDNF levels, and neonatal neurobehavioral outcomes. As far as we know, some researchers have developed drugs to elevate peripheral BDNF levels. Exploring the potential use of such drugs to enhance the neurodevelopmental behavioral abilities of the offspring of mothers with SCH is an exciting direction for further investigation (31).

### Limitations

This study has several limitations. Firstly, due to difficulty in follow-up, we could not evaluate newborns at 14 and 28 days after birth. Therefore, NBNA scoring was only conducted immediately after delivery, limiting our ability to assess whether the observed neurobehavioral outcomes persist or evolve over time. Secondly, although some studies have found a correlation between BDNF levels in the brain and peripheral blood, it remains uncertain whether alterations in peripheral blood BDNF levels directly correspond to modifications in central nervous system BDNF levels. Thirdly, this study was conducted with a limited sample size in a single center, which may not fully represent broader populations with diverse socio-economic, ethnic, or geographic backgrounds. Potential confounding factors, such as maternal diet, stress, and socioeconomic status, were also not accounted for in this study due to data unavailability. Finally, while BDNF screening shows potential as an early biomarker for neurodevelopmental risks, its clinical application faces ethical and practical challenges, including cost, accessibility, and the need for further validation in diverse populations. Future studies should incorporate multi-center designs, more diverse populations, and longitudinal approaches to address these limitations and enhance the generalizability and applicability of the findings.

## Conclusion

The umbilical cord blood brain-derived neurotrophic factor (BDNF) levels in pregnant women with subclinical hypothyroidism (SCH) are positively correlated with newborns’ behavioral and neurological abilities.

## Data Availability

The raw data supporting the conclusions of this article will be made available by the authors, without undue reservation.
